# Leveraging social media for resident training in developing countries: A case study of Libya

**DOI:** 10.17305/bb.2024.10806

**Published:** 2024-07-11

**Authors:** Faysal Al-Ghoula, Dimitrios K Kantas, Lucrezia Rovati, Ala Eddin Sagar, Mohammed Megri, Anas Zarmouh, Cameron G Gmehlin, Mohamed Ghit Benlamin, Tarik Ngab, Ognjen Gajic

**Affiliations:** 1 Division of Pulmonary and Critical Care Medicine, Mayo Clinic, Rochester, USA; 2 School of Medicine and Surgery, University of Milano-Bicocca, Milan, Italy; 3 Division of Internal Medicine, King Faisal Specialist Hospital and Research Centre, Madinah, Saudi Arabia; 4 Pulmonary and Critical Care Medicine, Marshall University Joan C. Edwards School of Medicine, Huntington, USA; 5 Department of Critical Care Medicine, Tripoli University Hospital, Tripoli, Libya; 6 Department of Internal Medicine, Mayo Clinic, Rochester, USA; 7 Emergency Department, Riverside Community Hospital, California, USA

**Keywords:** Remote training, intensive care unit (ICU), social media, Libya, developing countries.

## Abstract

Social media platforms have emerged as invaluable tools for remote training in resource-constrained countries. This study presents the development, implementation, and preliminary assessment of a remote intensive care unit (ICU) training program conducted in Libya utilizing social media platforms. This educational initiative was based on the Checklist for Early Recognition and Treatment of Acute Illness and iNjury (CERTAIN) program, targeting Libyan resident physicians. The initiative comprised a series of live-streamed pulmonary/critical care lectures and asynchronous discussions of clinical cases via a private messaging chat. Participant engagement, satisfaction, and learning outcomes were evaluated using social media analytics and surveys. A total of 323 learners joined the Libyan ICU training program chat group, and two tele-education sessions were broadcast, accumulating a total of 749 views. The majority (72.6%) of learners had graduated from medical school within the past five years and were in residency training. Clinical cases and learning materials were shared through 2991 messages in the chat group. Learners’ objective and subjective clinical knowledge improved after each tele-education session, and 88% of survey respondents rated the remote ICU training program as excellent. This study highlights the potential of using widely available social media platforms for remote ICU education in resource-limited settings.

## Introduction

The COVID-19 pandemic significantly increased the demand for critical care physicians while also exposing gaps in intensive care unit (ICU) training in many developing countries [1, 2]. During the COVID-19 pandemic, Libyian hospitals often reported inadequate numbers of trained intensive care physicians to fully staff ICUs and provide optimal patient care [3]. In Libya, limited access to educational resources, scarcity of trained medical educators, and lack of a standardized teaching system hamper the transfer of specialized knowledge and skills to the next generation of critical care clinicians [4–6]. Social media platforms and electronic classrooms have emerged as invaluable tools for remote ICU training in low, middle, and high-income countries alike during the COVID-19 pandemic, offering a multitude of advantages and benefits [7, 8]. These platforms provide accessible, cost-effective, and flexible avenues for healthcare professionals to access up-to-date medical knowledge and engage with subspecialty experts from around the world [9]. By fostering collaboration, networking, and enabling real-time interactions, social media platforms empower healthcare professionals in resource-limited settings to enhance their skills and deliver high-quality care to patients [10, 11].

In this article, we present a remote ICU training program conducted in Libya utilizing widely available social media platforms. The target group was resident physicians at the early stages of their careers. The aim of this study was to assess participant engagement and satisfaction with remote ICU training and to explore its impact on learning outcomes.

## Materials and methods

**Figure 1.**
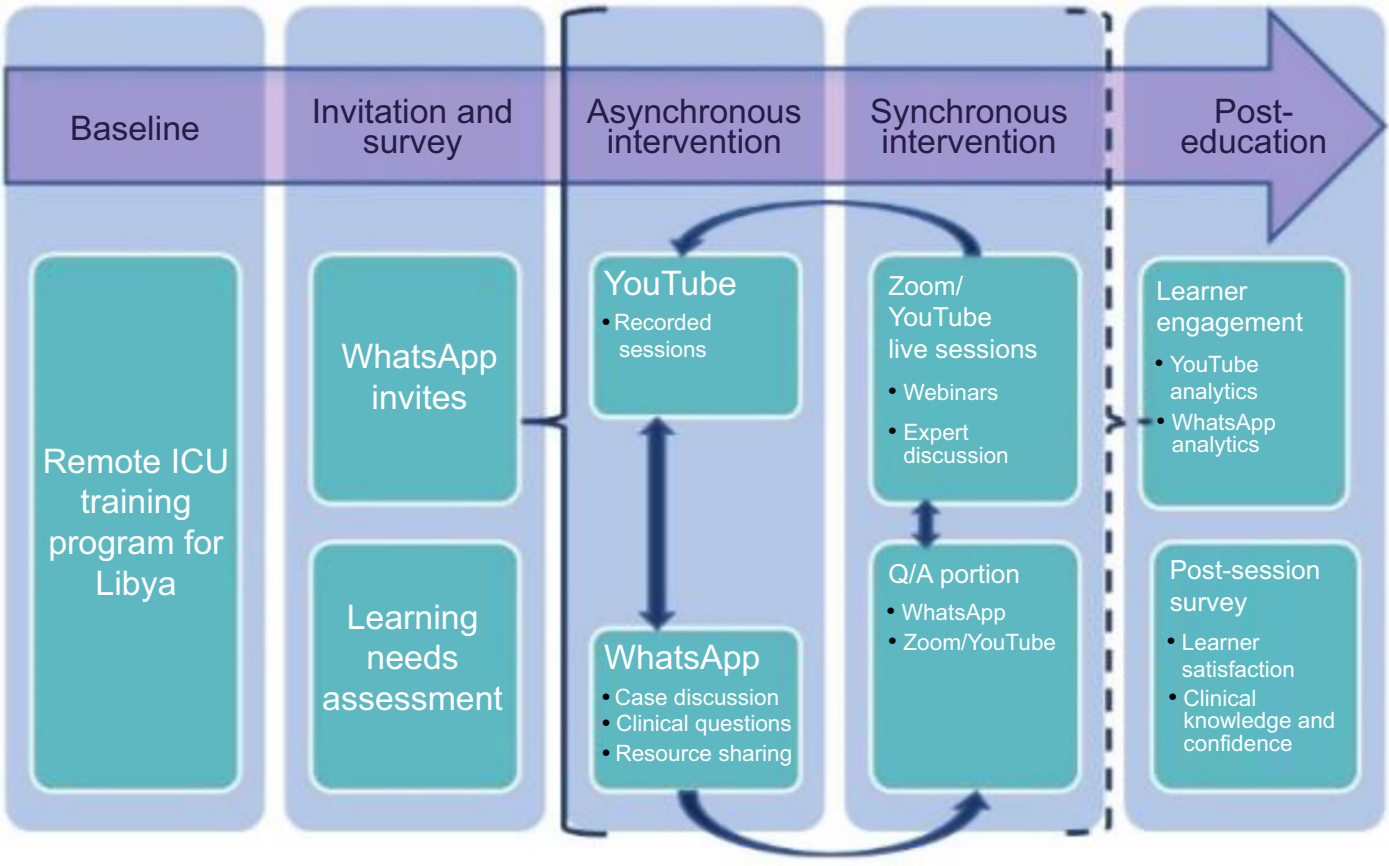
**Overview of the remote ICU training program for Libya**. ICU: Intensive care unit.

The remote ICU training program for Libya was based on the Checklist for Early Recognition and Treatment of Acute Illness and iNjury (CERTAIN) program, a global ICU best practices, education, and quality improvement initiative [12, 13]. This study evaluated the preliminary data collected during the first two months of the Libyan ICU training program, from April 4, 2023, to June 4, 2023 (Figure 1).

A training team was established by recruiting physicians from Libya who had completed their pulmonary and critical care training in the United States, UK, and South Africa, as well as local experts familiar with the Libyan healthcare context. Junior residents from Libya were invited to participate in the program using various channels, including announcements from professional societies, Facebook^®^ advertisements, and personal connections. The primary communication method between the training team and the participants was a dedicated, secure social media chat established on WhatsApp^®^ (Meta).

**Figure S1.**
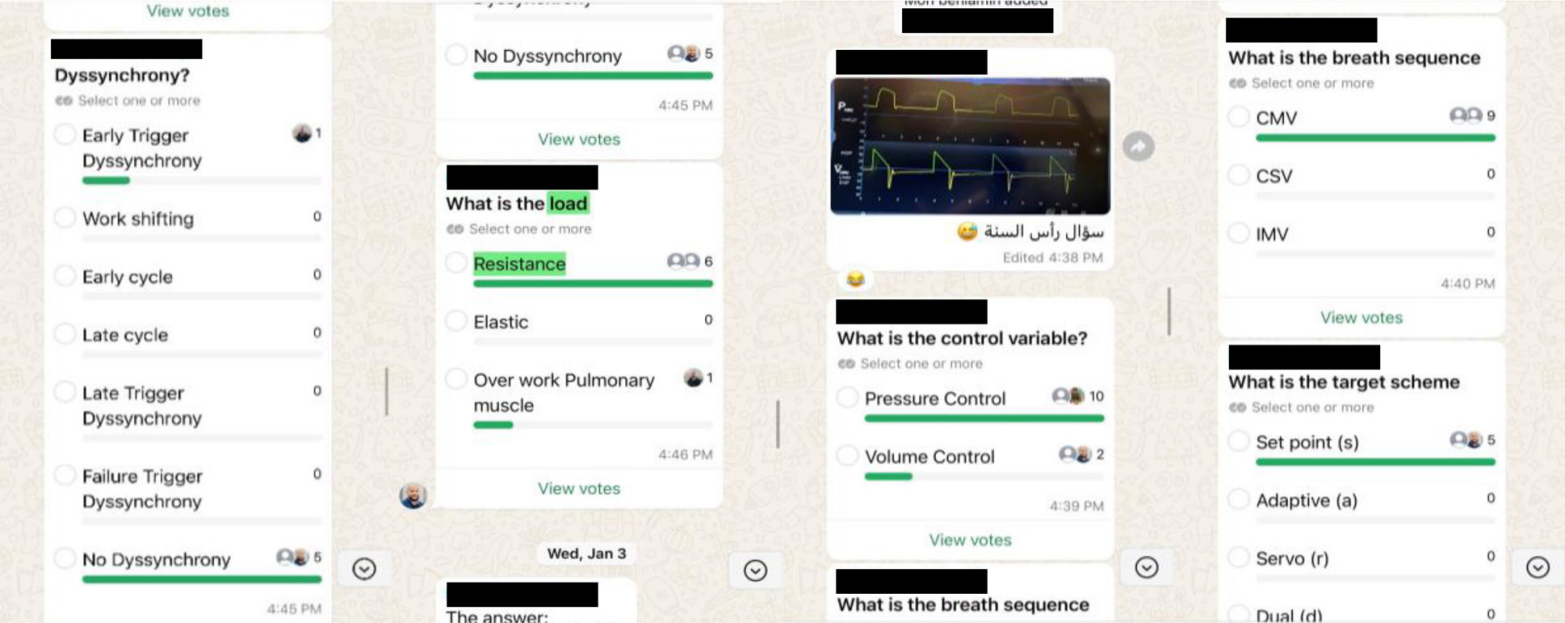
Example WhatsApp conversation.

Participants were encouraged to discuss challenging clinical cases via the private group chat using a standardized format (Figure S1). Polls were created to generate a differential diagnosis list for each case based on participant responses. Detailed explanations, guidelines, and other references were provided by the faculty through the private group chat to enhance learning. A formal tele-education curriculum consisting of a series of 45-min lectures on high-yield topics in pulmonary and critical care medicine was delivered using the Zoom^®^ videoconferencing platform (Zoom Video Communication). The sessions were livestreamed to a YouTube channel and the recordings were made available to allow asynchronous viewing. During the sessions, discussions between experts from Libya and abroad were conducted, and learners were able to ask questions. The involvement of local experts ensured the evaluation of recommendation applicability within the Libyan healthcare system, considering local challenges and resource availability.

**Figure S2.**
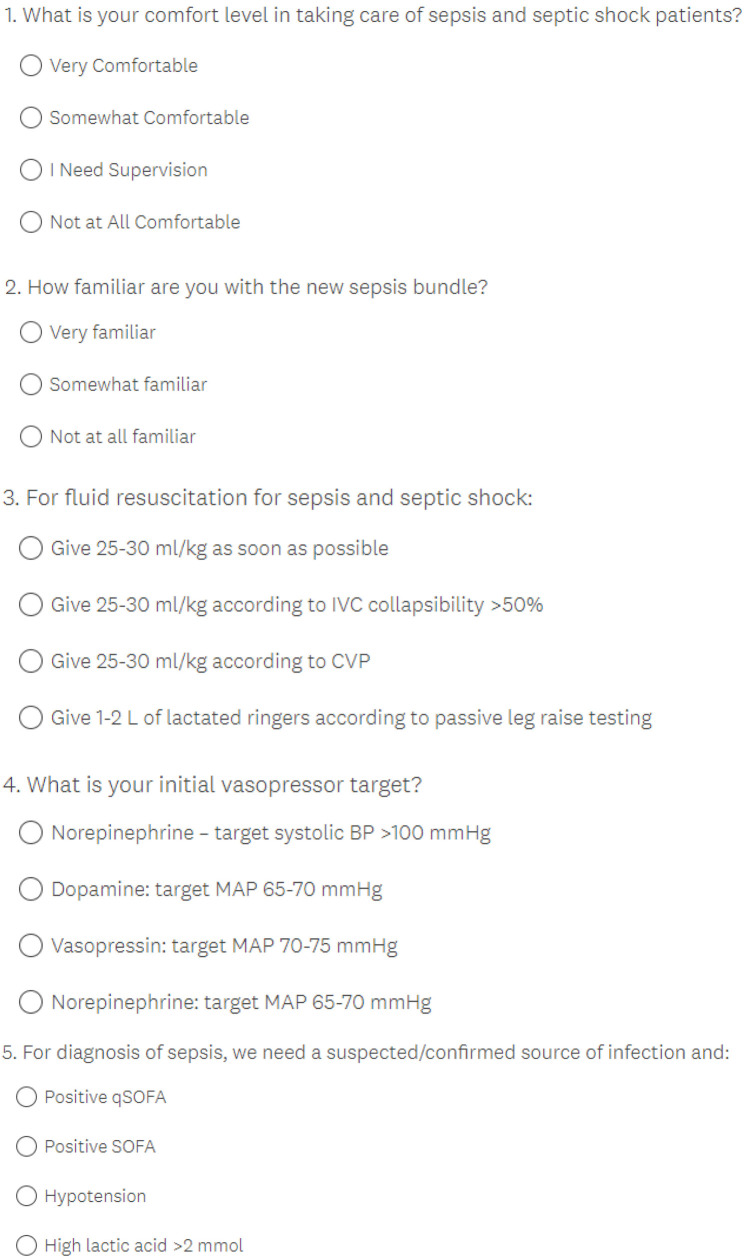
Pre/post sepsis/septic shock session survey.

Participant engagement, satisfaction, clinical knowledge, and confidence were evaluated using Survey Monkey polls conducted before and after each tele-education session and through analysis of WhatsApp and YouTube analytics (Figure S2). Descriptive statistics were reported as numbers with percentages or means with standard deviations (SD) as appropriate. Tables were created using Microsoft^®^ Excel, and figures were created using Prism^®^. Due to the nature of this study, IRB approval was waived and consent forms were not required.

**Table 1:** Participant characteristics

**Survey question**	**Responses, No. (%)** ***n* ═ 83**
1. When did you graduate from medical school?	
I am still a student	2 (2.4)
<2 years	19 (22.9)
2–5 years	42 (50.6)
>5 years	20 (24.1)
2. What is your current level of training?	
Medical student	3 (3.6)
Intern	20 (24.1)
Resident	42 (50.6)
Registrar	13 (15.7)
Not a trainee	5 (6.0)
3. In which hospital do you practice?	
Tertiary-care center	41 (49.4)
Secondary-care center	34 (41.0)
Private clinic	7 (8.4)
Not currently practicing	1 (1.2)
4. In which ward do you practice?	
ICU	48 (57.8)
Medical floor	19 (22.9)
Emergency department	14 (16.9)
Outpatient clinic	2 (2.4)

ICU: Intensive care unit.

## Results

A total of 19 educators participated in this initiative. The messaging group was established on April 4, 2023, and by June 4, 2023, 323 learners had joined. The majority of participants had graduated from medical school within the last five years (73.5%), were resident physicians (50.6%), and practiced in tertiary-care centers (49.4%) (Table 1). In the first two months of the initiative, 2991 messages were exchanged in the chat group, including 270 images, 243 audio messages, 90 videos, 33 PDF attachments, and 70 links to openly available online educational resources, such as scientific articles, books, and guidelines. Twenty-four unique participants actively contributed to the training program by sharing challenging cases, and 40 polls were used to collect responses from other learners on possible differential diagnoses and management strategies. The main topics discussed were lung mechanics, ventilator management, and ventilator dyssynchrony. The faculty coordinated the interactive case discussions and provided additional expert insights and scientific references.

**Figure 2.**
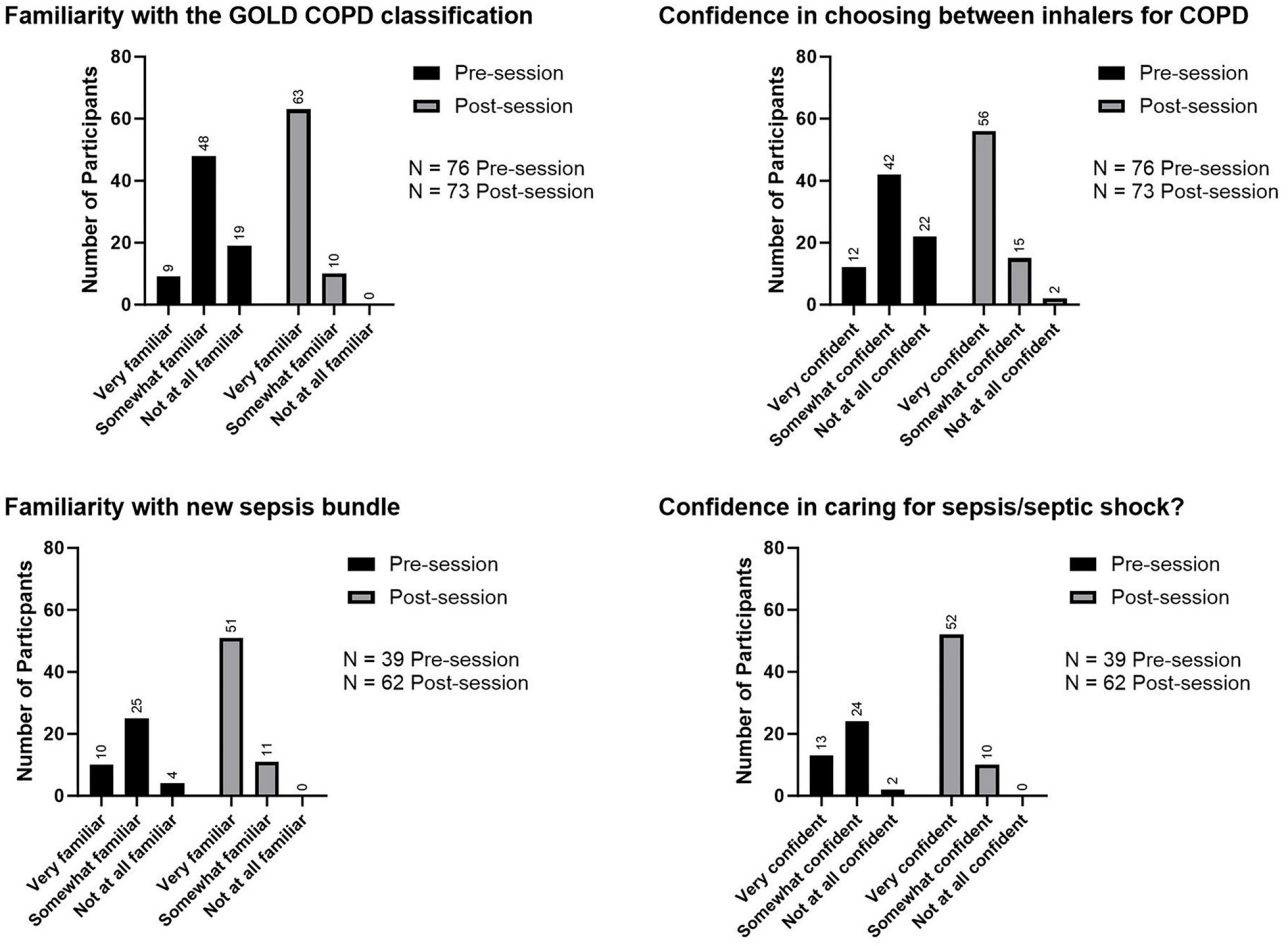
**Self-reported learning outcomes of tele-education sessions**. COPD: Chronic obstructive pulmonary diseases.

The first two tele-education sessions were live-streamed on April 30, 2022, and on May 15, 2022. The topics discussed during these initial lectures were chronic obstructive pulmonary diseases (COPD) and sepsis. One hundred participants attended the live stream of the first session on Zoom, which had 384 additional views on YouTube. Attendance for the second session consisted of 82 participants on Zoom with 183 total views on YouTube. Four questions to assess participants’ knowledge of the diagnosis and treatment of COPD and sepsis were asked before and after the sessions, and the proportion of correct answers increased from an average of 51% (SD 23.8%) before the sessions to an average of 84% (SD 11.8%) after the sessions. Surveys conducted before and after each tele-education session demonstrated an improvement in learners’ self-reported clinical knowledge and confidence (Figure 2).

On May 22, 2023, a survey to assess overall participant satisfaction was shared in the chat group. Of the 83 participants who answered the survey (response rate 27%), 88% rated the Libyan remote ICU training program as excellent, and the majority reported that this initiative had an impact on their medical knowledge and clinical practice (Table 2).

**Table 2:** Program evaluation by participants

**Survey question**	**Responses, No. (%)** ***n* ═ 83**
1. Has this initiative enhanced your medical knowledge?	
A lot	57 (68.7)
A moderate amount	26 (31.3)
Not at all	0 (0)
2. Has your practice been influenced by what you learned from this initiative?	
A lot	46 (55.4)
A moderate amount	34 (41.0)
Not at all	3 (3.6)
3. How would you rate the overall content of this initiative?	
Excellent	73 (88.0)
Too basic	5 (6.0)
Too advanced	5 (6.0)

## Discussion

This study highlights the potential of using social media platforms as effective tools for medical education in resource-constrained settings by allowing easier access to evidence-based educational and training opportunities. Preliminary data from the Libyan remote ICU training program show that residents and junior physicians engaged with the initiative and were satisfied with its content. Moreover, exploratory objective and subjective learning outcomes suggest that the program led to an improvement in participants’ self-reported clinical knowledge and confidence.

During the COVID-19 pandemic, social media platforms were used to rapidly create international healthcare collaborations for remote medical education and clinical support [8, 14–17]. These platforms are easily accessible and widely available on smartphones, making them an effective and cost-efficient tool for disseminating medical knowledge to clinicians working in resource-limited settings [18]. This is particularly advantageous for trainees in highly specialized fields who often have to resort to supplementing their knowledge using informal resources [19]. Programs that leverage social media like the Libyan remote ICU training program can provide participants with reliable, up-to-date clinical information without the use of complex technological infrastructure [20, 21].

A novel aspect of our study was the use of WhatsApp^®^ to foster discussion between learners across a wide range of institutions and educational levels. The use of secure instant messaging platforms allowed participants to select topics that addressed their immediate needs while maintaining engagement, maximizing learning outcomes, and ensuring the practical applicability of acquired knowledge to clinical practice. Prior studies examining the usefulness of secure instant messaging platforms like WhatsApp in an educational context have mainly focused on its use in US fellowship training [22, 23]. Participation rates were higher in those studies than the rates we observed; however, participation was likely buoyed by the setting (single center) and familiarity among the participants [24]. In contrast, participants in the Libyia ICU training program came from a diverse array of institutions, which may have deterred viewers from engaging in discussions or asking questions.

Another noteworthy aspect of our educational program was the incorporation of local educators who had firsthand knowledge of the resources and challenges faced by Libyan physicians. This approach ensured that the educational content was relevant and realistic, offering practical solutions to common problems. It is also worth noting that both educators and learners were young physicians familiar with new technologies, which might have further contributed to the high level of engagement and satisfaction observed in this study. 

Our study has several limitations. The self-selection of participants introduces the potential for selection bias, as those who volunteered may already have a higher interest in critical care education. Additionally, the reliance on self-reported data for most of the preliminary learning outcomes assessed introduces the risk of response bias and subjective interpretations. Finally, despite the high total number of participants, only a few learners actively contributed to the program by sharing challenging cases and engaging in discussion with the faculty. This is reflected by the low-response rate achieved in pre- and post-session surveys and the relatively limited list of topics discussed in the group chat. There are likely several factors contributing to this observation. First, participants may have felt vulnerable contributing, given their interactions were visible to a broad audience. Another explanation for low overall participation is that passive consumption is prevalent on social media platforms, where many users simply browse content without actively participating, thus creating a false impression of low engagement. In the future, we plan to continue this initiative by further promoting active engagement of the participants by using the communities feature in WhatsApp to allow individuals to browse and contribute to chats that they find interesting, and by assessing how this program might improve patient-centered outcomes.

## Conclusion

This case study demonstrates the feasibility of providing remote critical care education using widely available social media platforms. The program, which was specifically targeted to junior physicians, led to high participant engagement and satisfaction. Future studies will focus on broadening the range of topics discussed via social media, improving participation, and assessing whether the virtual training program can impact clinical outcomes.

## Supplemental data
